# Nutrition in Pediatric Extracorporeal Membrane Oxygenation: A Narrative Review

**DOI:** 10.3389/fnut.2021.666464

**Published:** 2021-08-02

**Authors:** Theresa S. W. Toh, Chengsi Ong, Yee Hui Mok, Palen Mallory, Ira M. Cheifetz, Jan Hau Lee

**Affiliations:** ^1^Department of Pediatric Medicine, KK Women's and Children's Hospital, Singapore, Singapore; ^2^Department of Nutrition and Dietetics, KK Women's and Children's Hospital, Singapore, Singapore; ^3^Children's Intensive Care Unit, Department of Pediatric Subspecialties, KK Women's and Children's Hospital, Singapore, Singapore; ^4^Division of Pediatric Critical Care Medicine, Duke University School of Medicine, Durham, NC, United States; ^5^University Hospitals Rainbow Babies and Children's Hospital, Cleveland, OH, United States; ^6^Duke-National University of Singapore Medical School, Singapore, Singapore

**Keywords:** energy expenditure, enteral nutrition, extracorporeal membrane oxygenation, nutritional adequacy, mortality, parenteral nutrition, pediatric intensive care unit

## Abstract

Extracorporeal membrane oxygenation (ECMO) support is increasingly utilized in quaternary pediatric intensive care units. Metabolic derangements and altered nutritional requirements are common in critically ill children supported on ECMO. However, there remains no consensus on the optimal approach to the prescription of nutrition in these patients. This narrative review aims to summarize the current medical literature on various aspects of nutrition support in pediatric patients on ECMO. These include: (1) nutritional adequacy, (2) pros and cons of feeding on ECMO, (3) enteral vs. parenteral nutrition, and (4) proposed recommendations and future directions for research in this area.

## Introduction

Extracorporeal membrane oxygenation (ECMO) has emerged as a global standard of care in the last two decades either as advanced life support in potentially reversible causes of cardiopulmonary collapse or as a bridge to heart and/or lung transplant (1, 2).

Nutrition support is an important component of daily clinical management in pediatric patients on ECMO. Despite the advances made in ECMO strategies and techniques, nutritional adequacy in patients on ECMO remains suboptimal (3). Little is known of optimal nutrient delivery practices in this heterogeneous group of patients, in particular about energy requirements and safety for enteral feeding. The provision of nutrition in children supported on ECMO has proved challenging because of the lack of a feasible method to estimate nutritional requirements, concurrent use of inotropes and concerns for gut ischemic injury, and lack of robust data to guide nutritional practices. This is further compounded by altered energy requirements and metabolic derangements in this critically ill population (4). Deleterious effects from overfeeding and underfeeding are well-documented. Malnutrition is reported to be associated with poor wound healing (5), loss of myocardial mass (6), and increased mortality (7), whilst excess nutrition has been found to be associated with steatosis and liver dysfunction (8). As such, optimization of nutrition is vital in the management of pediatric patients on ECMO.

This review aims to examine the following important aspects of nutrition management in children supported on ECMO: (1) nutritional adequacy; (2) timing of initiation; (3) enteral nutrition (EN) vs. parenteral nutrition (PN); (4) challenges faced; and (5) recommendations. For this narrative review, we searched the MEDLINE, PubMed, and Cochrane databases using the keywords “pediatric,” “enteral nutrition,” “parenteral nutrition,” and “extracorporeal membrane oxygenation.”

## Nutritional Adequacy

### Nutritional Requirement

Total energy expenditure (TEE) in a pediatric patient is comprised of resting energy expenditure (REE), energy to maintain metabolic function, and energy stored in new tissue for growth. Whilst the common opinion is that patients on ECMO are in a hypermetabolic state from their inflammatory burden, underlying illness, and catecholamine release to support the failing circulation (9), concurrent mitigating factors potentially reduce energy requirements for physical activity and thermoregulation (e.g., use of pharmacologic paralysis/sedation and cooling). Thus, a child supported on ECMO may not necessarily have an increased TEE. This is corroborated by studies demonstrating a decline in TEE and an overall hypometabolic state in infants post-cardiac surgery (10, 11). One study assessed TEE *via* respiratory mass spectroscopy in 17 infants post-Norwood surgery. These investigators found an initial high TEE that declined rapidly in the first 8 h and remained stable in this hypometabolic state for the next 48 h (10). Another study calculated the metabolic state of 30 children post-Fontan surgery using measured REE [*via* indirect calorimetry (IC)] as a ratio of estimated REE [using the World Health Organization (WHO) equation] (12). The study found that most patients were hypometabolic (33%) or normometabolic (30%) instead of hypermetabolic as often predicted (10, 11). While short-term exposure to cardiopulmonary bypass in congenital heart surgery and the stress associated with surgical interventions may not be fully applicable to children supported on ECMO, these findings provide rationale for the need for studies to examine this in children supported on ECMO. Indeed, with ECMO for cardiac failure accounting for up to 45% of pediatric ECMO runs annually (13), such studies would be important to better understand the TEE in these children. Further, compounding the assessment of nutritional requirement is the baseline nutritional status of the child which will invariably be affected by premorbid conditions. This leads to significant heterogeneity in nutritional requirements, emphasizing the need for an individualized approach for accurate assessment and delivery of nutrition.

TEE in pediatric patients supported on ECMO is not well-studied or understood. Few studies have directly assessed TEE on ECMO due largely to lack of available standardized measurement tools and the invasive nature of devices (e.g., direct calorimetry, IC, doubly labeled water technique). While standard equations (e.g., Schofield equation) are often used to estimate REE and determine caloric needs in critically ill children, there is a need to be cognizant that predictive equations are derived from healthy subjects which may lead to under or overestimation of caloric requirements in critically ill children (11, 14).

Certain investigators have advocated for indirect calorimetry to accurately assess energy needs of critically ill children to deliver appropriate energy requirements (15). The American Society of Parenteral and Enteral Nutrition (ASPEN) clinical guidelines for critically ill children echo this similar need for accurate assessment of TEE using IC, particularly in patients with metabolic alterations (e.g., neurologic trauma, thermal injury, hypermetabolic state) and in those who are malnourished (16). To date, no pediatric study has reported the use of IC in ECMO. Challenges with using IC in ECMO stem from the presence of two gas exchange systems that need to be considered—patient's native lung and membrane oxygenator. Traditional indirect calorimetry only measures gas exchange of the patient's native lung, resulting in an underestimation of total oxygen consumption and carbon dioxide production in ECMO patients. One adult study by De Waele et al. (17) employed use of IC by sampling gas exchange at both the ventilator and membrane oxygenator. These authors found median REE in adult ECMO patients to be 18 kcal/kg/day (IQR 15 – 27), lower than traditional energy estimations of 25 kcal/kg/day (18). Respiratory mass spectroscopy has been used as a feasible alternative to estimate TEE in pediatric patients on ECMO (19). Respiratory mass spectroscopy measures oxygen consumption (VO_2_) *via* an inert gas dilution method wherein mass flow of a marker gas is injected into expired gas upstream with the resulting gas composition downstream used to deduce the mass flow of all components. As such, estimating TEE using respiratory mass spectroscopy involves a VO_2_ calculation from the ECMO and ventilator circuit in sequential fashion (20). In a study utilizing respiratory mass spectroscopy, the TEE of children on ECMO aged 0.3 to 36 months was estimated to be 40–46 kcal/kg/day (19). This appears to most closely resemble basal metabolic rate without the use of any stress factors, contradicting traditional thought that critical illness requiring ECMO support is associated with a hypermetabolic state. A caveat to this observation is the significant lack of a standardized modality to accurately assess TEE. Alternative body composition assessment methods to estimate TEE including bioelectrical impedance analysis (21), air displacement plethysmography (22), and dilution techniques with stable isotopes have been explored in small pediatric studies but accuracy is confounded by changes in fluid status (23, 24). These techniques can be potentially refined and investigated for use in children supported on ECMO. Indeed, current guidelines lack official consensus on TEE or caloric requirements in pediatric ECMO.

At present, insufficient data are available to provide evidence-based recommendations for protein intake for pediatric ECMO patients. Children with critical illness experience increased whole body protein degradation during stress response resulting in a net negative protein and nitrogen balance that manifests as muscle wasting and immune dysfunction (16). Neonates have been shown to have 25% higher protein degradation post-surgery (trachea-esophageal fistula, small/large bowel resection, omphalocele repair) and a 100% increase in urinary nitrogen excretion with bacteremia (25, 26). According to the 2017 ASPEN clinical guidelines, recommended minimum protein requirement for critically ill children is 1.5 g/kg/day (27) whilst for the neonatal population this can be as high as 3 g/kg/day (28). Most centers target a protein goal of 2–3 g/kg/day in neonatal and pediatric populations (29, 30). Adequate protein delivery is associated with increased ventilator-free days and reduced mortality (31) in one retrospective study of 54 pediatric patients demonstrating that protein intake of >80% prescribed by day 7 of ECMO was also associated with decreased ECMO duration (*p* < 0.005) (32). One multicenter study of 1,245 mechanically ventilated patients revealed a dose dependent relationship between a higher percentage of protein delivery and lower mortality (33). As most pediatric patients requiring ECMO are invariably mechanically ventilated, it may be reasonable to extrapolate this protein delivery practice to these patients. Parameters such as blood urea nitrogen, urinary nitrogen, plasma amino acids (e.g., leucine), and muscle thickness have been employed as feasible measures of protein adequacy in small neonatal (34) and adult (35) studies. To date, no study has measured protein adequacy in pediatric patients on ECMO.

The overall impact of ECMO on micronutrient alterations and homeostasis is not well-studied. A previous *ex vivo* ECMO model demonstrated circuit losses of essential amino acid isoleucine, vitamin A, and vitamin E *via* postulated mechanisms of instability, degradation, circuit sequestration, or oxidation in the ECMO circuit (36). Vitamin A, in particular, is light-sensitive and prone to photolysis even in fat emulsion and light-protected bags (37). A single-center retrospective study of 78 pediatric patients on ECMO found that calcium derangements occur frequently and are associated with increased ECMO duration, length of ICU stay, and hospital stay (38).

Additionally, for ECMO patients requiring continuous renal replacement therapy (CRRT), micronutrient losses, in particular vitamins B1, B6, B9, C, and trace elements (especially selenium), are augmented (39, 40). A narrative review of published literature on nutritional therapy during CRRT in critically ill pediatric patients found that the effluent (removed fluid) extracted *via* the convection process contains the expected toxic metabolites and also proteins, amino acids, vitamins, and trace elements (40). However, pediatric studies on detailed vitamin removal rates are scarce with the majority of findings being extrapolated from adult studies wherein vitamin losses were estimated from effluent flow rate (41). Multiple micronutrient deficiencies are postulated to affect the body's response to oxidative stress in critically ill patients leading to immune dysfunction, though, randomized controlled trials conducted in adult patients with micronutrient deficiency associated with severe oxidative stress showed that supplementation of micronutrients failed to improve outcomes of mortality, length of stay, or infection (42). There remains a paucity of data on micronutrient homeostasis and management in the critically ill pediatric population, and this continues to be a key area for further research.

## Timing Of Initiation Of Nutritional Support

Provided there are no contraindications to feeding, centers generally advocate for EN to be initiated within 24 to 48 h of admission to the pediatric intensive care unit (PICU) (16). However, in patients on ECMO, there are many perceived barriers to initiation of EN; these include mode of extracorporeal life support, underlying diagnosis, recent period of potential hypoxic-ischemic injury, and vasopressor status (43). A retrospective review of 149 children requiring ECMO found differences in cumulative energy deficits over the first week in those who received late PN (>48 h of admission) compared to those who received early PN (within 48 h of admission)—energy deficit was reported at 176 ± 90 vs. 220 ± 93 kcal/kg for early PN and late PN cohorts, whilst cumulative protein deficit was reported at 5.1 ± 3.3 and 7.9 ± 3.8 g/kg, respectively (44). However, recent studies in the PICU setting have cast concerns on early PN in critically ill children. A randomized controlled trial conducted by Fivez et al. of 1,440 critically ill children (PePANIC trial) demonstrated that late PN (started on day 8 of admission) was associated with a shorter duration of mechanical ventilation [4.4 ± 0.3 vs. 6.4 ± 0.7 days; *p* = 0.001], lower requirement for CRRT [2.5 vs. 3.6%; *p* = 0.04], and shorter ICU stay [6.5 ± 0.4 vs. 9.2 ± 0.8 days; *p* < 0.001] when compared to early PN (within 24 h of admission). There was otherwise no difference in mortality between the two groups (45). Two-year follow-up in this same cohort showed that late PN did not affect survival, neurocognitive development, or health status (46). However, the findings of the PePANIC trial should be interpreted in the context of the study's limitations. These include the lack of blinding of patients, parents, and ICU health care providers on treatment assignments. Additionally, in the follow-up period, the young age of the majority of the patients in the study cohort precluded complete and reliable results for certain neurocognitive testing. Furthermore, in the late PN group, a significant proportion of patients did not receive PN. More definitive studies are needed to ascertain if these findings of late PN and improved outcomes are consistent in pediatric patients requiring ECMO.

## Enteral vs. Parenteral Nutrition

Many barriers to early EN have been identified in patients supported on ECMO. These include the need for vasopressors, use of veno-arterial (VA) ECMO, and physician fear of intestinal ischemic injury and presence of congenital diaphragmatic hernia (43). Whilst these are not strict contraindications, they pose as significant challenges in initiating early EN. As such, in an overwhelming majority of children supported on ECMO, EN alone is insufficient to meet the energy needs and PN is often required. ECMO is often used in children requiring high inotropic support and the use of EN may be of concern in these children. In the critically ill pediatric cohort on high inotropic support, enteral feeding appears to be safe with no significant gastrointestinal complications and has a trend toward lower mortality (47, 48). However, safety of feeding in ECMO patients on vasopressors is not well-studied. A small retrospective review of 52 ECMO patients on various combinations of inotropes (no vasoactive inotropic score available) reported no adverse events with enteral feeding (49). Another retrospective study demonstrated an overall low vasoactive inotropic score (VIS) by day 5 of ECMO indicating that there was not an abundant use of vasopressors in this cohort of patients in the first place potentially allowing for safe initiation of enteral feeding (50). Whilst animal studies suggest reduced gut perfusion and gut barrier dysfunction on ECMO support (51), current published studies seem to favor early initiation of EN in children supported on ECMO. A retrospective single center study of 49 patients demonstrated that any EN on day 5 of ECMO was associated with greater survival to discharge compared to exclusive PN and no significant difference was noted in incidence of enteric bacterial infections or serious abdominal complications (50). Studies in the neonatal population on ECMO similarly demonstrate that EN is safe with no significant increase in abdominal complications (52, 53). A retrospective chart review of 77 neonates on ECMO showed that EN was only temporarily discontinued in 20.7% (*n* = 16/77) of the study cohort due to mild abdominal symptoms of gastric retention and perceived abdominal discomfort; however, no serious adverse events, such as necrotizing enterocolitis, were reported (52). Another neonatal study retrospectively analyzed 51 neonates on ECMO (16 EN vs. 35 PN) and showed that septic complications occurred with similar frequency in EN and PN groups, with no statistically significant difference in mortality rate (0 vs. 14%, *p* = 0.17) (53); however given the small study size, this will benefit from further research. One study demonstrated that in spite of compromised intestinal integrity (as measured by excretion percentages of lactulose, D-xylose, and 3-O-methyl-D-glucose *via* gas-liquid chromatography in urine samples) in neonates on ECMO, EN was well-tolerated with no adverse events of necrotizing enterocolitis observed (54). Compared to pediatric patients, the neonatal gut is generally more at risk for translocation of bacteria and ischemia (55). Thus, the demonstration of safety for EN in neonates on ECMO can potentially be extrapolated to pediatric ECMO patients, who may have an even greater margin of safety with EN, given a potential lower risk of translocation and ischemia. Nevertheless, pediatric studies specific to this area are needed.

## Current Practices and Challenges Faced

Multiple retrospective studies have assessed nutritional adequacy in pediatric ECMO patients. Overall nutritional adequacy is found to be suboptimal with significant challenges faced in initiation of EN as previously described. Single center pediatric ECMO studies have found the percentage of patients receiving >80% of target energy and protein requirements to be 25 and 18%, respectively, by day 3 of ECMO (56), and 65 and 61%, respectively, by day 7 of ECMO (44). In these studies, a higher vasoactive inotropic score (VIS) and use of VA ECMO were associated with lower nutritional adequacy and lower EN. The main barriers and challenges to feeding on ECMO remain to be physician fear of ischemic gut injury, use of vasopressors, and underlying diagnosis, in particular severe sepsis and surgical issues (43).

## Proposed Algorithm

**Figure 1:**
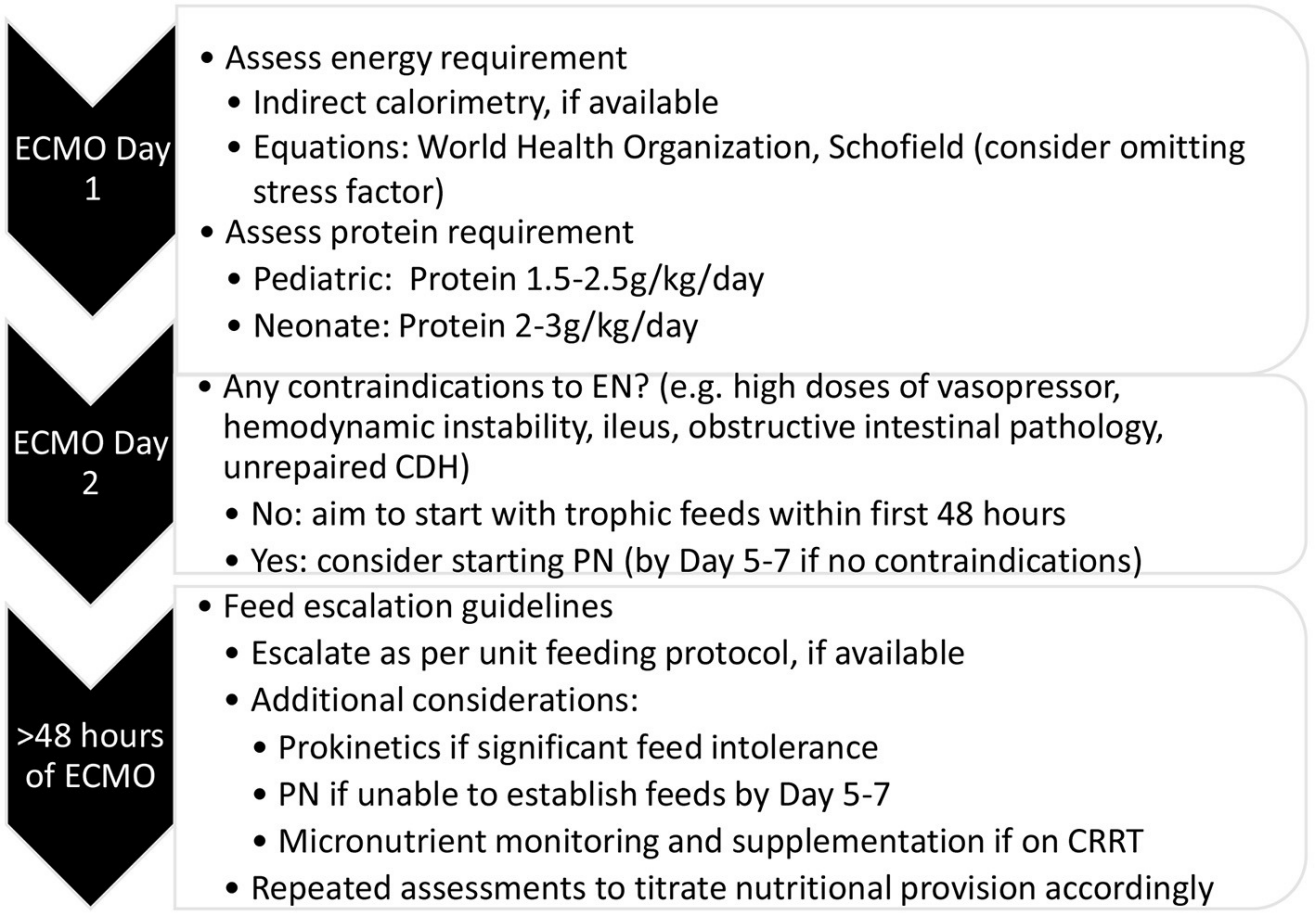
Proposed approach for nutritional support in pediatric extracorporeal membrane oxygenation. CDH, congenital diaphragmatic hernia; EN, enteral nutrition; PN, parenteral nutrition; CRRT, continuous renal replacement therapy.

Given the above evidence on determining nutritional requirement, timing of initiation, and route of nutrition delivery, we propose the following algorithm to consider for nutritional support in this vulnerable population (Figure 1). Whilst we acknowledge the paucity of robust evidence in the field of nutrition in pediatric ECMO, our review of the literature suggests the safety of early enteral feeding and need for accurate assessment of nutritional requirement.

### The Use of Prokinetics

Whilst there are no established data to support the use of prokinetic agents in improving nutritional adequacy in pediatric ECMO patients, centers have reported that feed intolerance contributes up to one third of feed interruptions in these patients (49, 56). Prokinetic agents are postulated to potentially reduce feed intolerance, and a study of 51 patients on VA ECMO found that prevalence of prokinetic use was up to 27% with no significant complications reported (56).

### Feeding Protocols

Feeding protocols are not the standard of care in the management of pediatric patients on ECMO and is used in a minority of centers (43). Several studies and a systematic review demonstrated that institution of a standardized feeding protocol in the critically ill pediatric patient can significantly reduce timing to attaining feeding goals and improve feed tolerance but these studies were not specific to ECMO patients (57, 58).

## Future Directions

A search of the clinicaltrials.gov, MEDLINE, and Cochrane databases did not reveal any registered ongoing clinical trials on outcomes related to nutrition in pediatric ECMO. In our narrative review, we included neonatal studies and other relevant adult studies, where applicable, but published literature remains limited especially for the pediatric and neonatal populations. Our review highlights the lack of standardization and availability of accurate modalities to estimate TEE and the lack of robust studies to ascertain optimal timing of nutrition initiation (for both EN and PN). The current state of research highlights the significant challenges in conducting nutrition research in this particular area. However, given the rapid increase in ECMO use, there is an urgent and critical need for future research to guide nutrient delivery practices. Future research in this patient population should focus on discovering an accurate and convenient method to measure TEE and protein adequacy, establishing the optimal route of administration of nutrition, and investigating both short- and long-term outcomes (e.g., neurocognitive and physical function) that may be modulated by nutrition interventions.

## Conclusion

Optimal nutrient delivery practices are still to be determined in pediatric ECMO patients. Barriers to delivery of optimal nutrition include lack of accurate nutrition assessment tools, concerns of ischemic gut injury with EN and emerging concerns of early PN on patient outcomes among critically ill children. Additional research is required to elucidate optimal timing of initiation, route of administration, and nutritional requirement of pediatric patients supported on ECMO.

## Author Contributions

TT and JHL contributed to conception and design of the review. TT wrote the first draft of the manuscript. CO, YHM, PM, IC, and JHL critically edited and contributed to the scientific content of the manuscript. All authors contributed to manuscript revision, read, and approved the submitted version.

## Conflict of Interest

The authors declare that the research was conducted in the absence of any commercial or financial relationships that could be construed as a potential conflict of interest.

## Publisher's Note

All claims expressed in this article are solely those of the authors and do not necessarily represent those of their affiliated organizations, or those of the publisher, the editors and the reviewers. Any product that may be evaluated in this article, or claim that may be made by its manufacturer, is not guaranteed or endorsed by the publisher.
